# Immune Memory in Aging: a Wide Perspective Covering Microbiota, Brain, Metabolism, and Epigenetics

**DOI:** 10.1007/s12016-021-08905-x

**Published:** 2021-12-15

**Authors:** Ozlem Bulut, Gizem Kilic, Jorge Domínguez-Andrés

**Affiliations:** 1 Department of Internal Medicine, Radboud University Medical Center, Geert Grooteplein Zuid 8, 6525 GA Nijmegen, Netherlands; 2 Radboud Institute for Molecular Life Sciences (RIMLS), Nijmegen, Netherlands

**Keywords:** Immune memory, Immunosenescence, Aging, Trained Immunity, Metabolism, Microbiota

## Abstract

Non-specific innate and antigen-specific adaptive immunological memories are vital evolutionary adaptations that confer long-lasting protection against a wide range of pathogens. Adaptive memory is established by memory T and B lymphocytes following the recognition of an antigen. On the other hand, innate immune memory, also called trained immunity, is imprinted in innate cells such as macrophages and natural killer cells through epigenetic and metabolic reprogramming. However, these mechanisms of memory generation and maintenance are compromised as organisms age. Almost all immune cell types, both mature cells and their progenitors, go through age-related changes concerning numbers and functions. The aging immune system renders the elderly highly susceptible to infections and incapable of mounting a proper immune response upon vaccinations. Besides the increased infectious burden, older individuals also have heightened risks of metabolic and neurodegenerative diseases, which have an immunological component. This review discusses how immune function, particularly the establishment and maintenance of innate and adaptive immunological memory, regulates and is regulated by epigenetics, metabolic processes, gut microbiota, and the central nervous system throughout life, with a focus on old age. We explain in-depth how epigenetics and cellular metabolism impact immune cell function and contribute or resist the aging process. Microbiota is intimately linked with the immune system of the human host, and therefore, plays an important role in immunological memory during both homeostasis and aging. The brain, which is not an immune-isolated organ despite former opinion, interacts with the peripheral immune cells, and the aging of both systems influences the health of each other. With all these in mind, we aimed to present a comprehensive view of the aging immune system and its consequences, especially in terms of immunological memory. The review also details the mechanisms of promising anti-aging interventions and highlights a few, namely, caloric restriction, physical exercise, metformin, and resveratrol, that impact multiple facets of the aging process, including the regulation of innate and adaptive immune memory. We propose that understanding aging as a complex phenomenon, with the immune system at the center role interacting with all the other tissues and systems, would allow for more effective anti-aging strategies.

Human beings, like all organisms, inevitably age and die. Even if science eventually cracked the code for immortality, that would not end the need to understand the mechanisms of aging and the efforts to slow or revert it. If anything, it will be even more critical to maintain the health of all cells and organs throughout a long life. Tackling aging is always a worthwhile effort to improve the quality of live for the middle-aged and elderly populations, especially since the human population over 60 years of age is expected to reach two billion by 2050 [1].

Infectious diseases of the elderly, especially in low-income countries, represent a significant social and economic burden. The immune system undergoes numerous changes as humans age, leaving older individuals more prone to disease [2]. The age-related dysregulations in the immune system are collectively referred to as “immunosenescence” and include accumulating tissue damage, a low-grade chronic systemic inflammation termed “inflammaging,” impaired immune cell function, inadequate response to vaccination, and increased vulnerability to infections [3].

The importance of immune memory has perhaps never been more evident than during the ongoing coronavirus disease 2019 (COVID-19) pandemic, which disproportionately affected the elderly population due to the altered functionality of their immune system [4]. Thanks to the outstanding collaborative effort of governments and scientists, 7 vaccines generating effective immune response and protection against the severe acute respiratory syndrome coronavirus 2 (SARS-CoV-2) have been authorized for emergency use by World Health Organization (WHO)–recognized authorities as of June 2021, and many more are in use with authorizations by national regulatory agencies [5]. Due to the increased vulnerability of the elderly, they are the priority group in COVID-19 vaccination rollouts.

Besides the morbidities caused by infection, the elderly also present an increased incidence of metabolic diseases such as type 2 diabetes and obesity [6], and neurodegenerative disorders such as Alzheimer’s and Parkinson’s diseases [7]. However, the development of these age-related conditions is not separated from their aging immunity. All systems and organs exchange signals with and are influenced by the immune system. Combining all the accumulating insights from different lines of research is critical to drawing up a comprehensive view of aging.

In this review focusing on immune memory, we first outline how memory is developed and maintained. Next, we delve into metabolic and epigenetic mechanisms, their roles in immune memory, how they change with age, and the implications for age-related pathologies. As two examples of the far-reaching impacts of an aging immune system, we highlight the interplay of immune memory with the gut microbiota and the brain. We end the review by presenting the current preventative and therapeutic strategies against aging, approaching from the alternative points of view of epigenetic modulation, metabolic intervention, microbiota reconstitution, and neuroprotection.

## Adaptive Immune Memory

Infections have been one of the primary selective forces throughout evolution, so immunological memory has evolved to ensure survival when an organism is exposed to a pathogen that it encountered before [8]. Until the discovery of non-specific innate immune memory in the last decade, the antigen-specific memory established by T and B lymphocytes have been getting all the credit for long-term protection against pathogens.

### T Cells: Thymus-Derived Troops of Immunity

Immunological memory against infections and tumors requires the intervention of T cells. T cells can recognize both self and non-self antigens through their T cell receptors (TCRs) and mount self-tolerance or immunological memory. Different subsets of T cells include naïve T cells that recognize new antigens and memory T cells that are formed upon former exposure to antigen and assure long-lasting immunity.

#### T Cell Development

T cells derive from the hematopoietic stem cells (HSCs) in the bone marrow but mature in the thymus. Most mature T cells reside in lymphoid tissues, but they are ubiquitously present throughout the body. After lymphoid progenitors migrate from bone marrow to the thymus, TCR gene rearrangement occurs, and CD4^+^ CD8^+^ double-positive cells expressing both co-receptors are generated. Then, these cells undergo positive selection based on TCR-antigen interactions and differentiate into naïve single positive CD4^+^ helper or CD8^+^ cytotoxic T cells, which are released into the periphery [9].

Most of our knowledge on T cell development originates from mouse studies. However, there are substantial differences between mice and humans. For instance, although the peripheral naïve T cell pool is almost exclusively provided by the thymus in mice, humans primarily sustain it by peripheral cell division [10].

When a naïve cell recognizes an antigen presented by antigen-presenting cells (APCs) such as dendritic cells (DCs) and macrophages, they proliferate and develop into effector cells that can clear the source of the antigen, likely a pathogen. A small portion of these effector cells later become memory cells to establish long-term immunity that can last multiple decades, while the rest die by apoptosis [11]. Early in life, before exposure to many antigens, naïve T cells constitute most of the T cell pool [12]. Meanwhile, regulatory T cells (Treg) are critical for the development of tolerance for innocuous antigens in the environment [13].

Around 5% of all adult CD4^+^ T cells are Tregs that are able to suppress the immune response [12]. Tregs are produced in the thymus but can also derive from peripheral naïve T cells by acquiring *Forkhead Box P3* (*FOXP3)* expression in response to environmental cues [13]. Recently, Tregs were shown to acquire memory characteristics, mostly against self-antigens, to prevent unwanted inflammation [14].

Memory T cells are divided into three subtypes which are central memory (TCM), effector memory (TEM), and stem cell memory (TSCM). Compared to TEMs, TCMs have more proliferation capacity and are closer to naïve T cells in gene expression profiles [15]. TEMs can perform effector functions such as cytokine production. TSCMs are a stem cell-like, less differentiated cell type with high self-renewal capacity and the ability to differentiate into effector T cells, TEMs, or TCMs [16]. Following TCR stimulation, they are able to secrete interferon gamma (IFN-γ) and interleukin 2 (IL-2). The long-lasting, multipotent TSCMs might help protect the organism against infections later in life when thymic output is low.

Although 90–95% of the effector T cells die after an infection resolves, a population of terminally differentiated effector cells regaining the naïve T cell marker CD45RA, termed TEMRA cells, remain in circulation. These senescent-like cells have defects in telomerase expression and proliferation; however, they are capable of cytokine production and cytotoxicity, unlike exhausted cells [17].

In many tissues such as lungs, intestines, and spleen, TEMs are the predominant T cell type [18, 19]. Moreover, discrete tissue-resident memory T cell populations (TRM) are identified with enhanced expression of adhesion markers and homing receptors, lower proliferative capacity, and higher production ability of pro-inflammatory and anti-inflammatory cytokines [20]. They can quickly react upon tissue injury or infection while also restricting the inflammatory damage. Establishing TRMs is a promising approach to consider in vaccine design, boosting and prolonging vaccine-mediated protection [21–24].

#### Effects of Aging on T Cells

Lineage differentiation dynamics of HSCs in the bone marrow are altered with age. They skew towards myeloid differentiation, leading to lower numbers of lymphoid cells in the elderly [25]. HSCs also accumulate DNA damage throughout life and differentiate into leukocytes with chronic DNA damage response [26]. This triggers cellular senescence, which contributes to chronic inflammation by inducing a senescence-associated secretory phenotype (SASP), impacting neighboring immune and non-immune cell types. Another way that DNA damage can contribute to inflammation is the activation of DNA-dependent protein kinase catalytic subunits (DNA-PKcs) that can promote NFκB and inflammasome activity [27, 28].

Involution of the thymus is one of the critical age-dependent changes in the immune system [29]. It is an evolutionarily conserved phenomenon in all vertebrates, starting before puberty, where the total mass, volume, and cellular content of the thymus shrink [30]. Thymic activity does not entirely cease, at least until the sixth decade of life, but thymopoiesis strikingly decreases with age [31, 32]. Thymic epithelial cells gradually lose the ability to produce IL-7, which is crucial to support thymopoiesis [33, 34]. Low thymic output in the elderly is associated with increased vulnerability to infections [35]. In a young adult, the thymus provides around 16% of the naïve T cell pool, the rest of which derives from peripheral proliferation [36]. In the elderly, this number falls below 1%, causing them to entirely rely on the proliferation of existing naïve T cells.

The decline in the number of naïve T cells and accumulation of terminally differentiated cells are two of the hallmarks of T cell aging [36]. CD4^+^ and CD8^+^ naïve cell pools, although more markedly for CD8^+^ T cells, contract in the elderly. Maintenance of naïve T cells through peripheral proliferation is more successful for CD4^+^ T cells, but CD8^+^ T cells are largely lost. Interestingly, while this is mostly the case in cytomegalovirus (CMV) + individuals in women, it is observed in men irrespective of the CMV status [37]. Also, CMV + individuals of both sexes have a higher proportion of late-differentiated senescent T cells than CMV individuals.

Chronic CMV infection affects most adults, with an 83% global seroprevalence rate [38]. Even though it usually does not cause active symptoms and is mainly unrecognized, CMV presence significantly shapes the T cell compartments and accelerates immunosenescence. Accumulation of terminally differentiated T cell types such as TEMs and TEMRAs occurs faster in CMV + individuals throughout their lifespan [39]. Expansion of CD8^+^ TEMRA cells is related to impaired antibody production upon influenza vaccination in the elderly [40]. Latent CMV infection is also associated with inadequate CD4^+^ T cell response against influenza antigens [41]. Moreover, CMV positivity is associated with a higher risk of all-cause mortality [42]. Of note, CMV + young adults displayed higher antibody responses to influenza vaccination, compared to CMV − young individuals [43]. In the early stages of the infection, CMV might be potentiating immune responses before the accumulation of CMV-induced senescent cells pass a certain threshold and causes functional impairments.

Not just the numbers but also the receptor diversity of naïve T cells are compromised in aged organisms. Naïve T cells of a young adult carry around 100 million different TCR sequences; however, this repertoire diversity is reduced up to tenfold in the elderly [44]. Moreover, memory T cells experience a narrowing of TCR repertoires [45], and the proliferative capacity of senescent T cells following TCR engagement is defective [46]. Activated CD8^+^ cells of elderly individuals also produce lower levels of cytotoxins such as granzyme B and perforin [47]. On the other hand, CD4^+^ naïve T cells of the elderly seem to maintain their differentiation and subsequent cytokine production capacities [48].

Lastly, differentiation of non-Treg cells into Tregs and proliferation of existing Tregs can maintain the Treg pools throughout life, despite reduced thymic output with aging. However, the balance between T cell subsets is altered: as in other T cell types, the naïve subset declines with age while memory Tregs increase [49].

### B Cells: Bone Marrow-Born Battlers

B cells are a vital part of the adaptive immune memory. They have several immunological functions, including antibody and cytokine production, antigen presentation, and regulation of T cell responses [50]. Most vaccines mainly target and rely on B cell activation by inducing long-lived plasma and memory B cell proliferation[51]. However, aging affects the functional capacity of existing B cell subsets drastically, which is evident from the susceptibility to diseases and poor vaccine responses [52].

#### B Cell Development

B cells continuously arise from the hematopoietic stem cells (HSCs) and develop in the bone marrow (BM) [53]. HSCs generate multipotent progenitors that eventually diverge to common lymphoid progenitors (CLPs). Certain environmental cues, transcription factors (TFs), cytokines, and chemokines lead CLPs to differentiate into B-cell lineage. Following differentiation, cells undergo a rearrangement in the variable regions of the immunoglobulin (Ig) genes and start to express B-cell receptors (BCRs) and IL-7 receptor (IL-7R) [54]. Each B cell has a unique BCR with a different specificity to antigens.

B cells that finish their developmental process in the bone marrow are called transitional (TR) B cells. They make 4% of all B lymphocytes in healthy individuals [55] and are found in several places, including the bone marrow, peripheral blood, and secondary lymphoid tissues. Transitional B cells become either marginal zone (MZ) or mature follicular (FO) cells partly based on the strength of their BCR signaling. Cells with more robust signaling tend to develop into follicular type, while weaker signaling drives them to be MZ cells [56]. FO B cells have a broad immunoglobulin repertoire and are located in the follicles close to T cell zones [57]. Therefore, they are suited for getting T-cell help and becoming short-lived plasma cells. On the other hand, MZ B cells can get activated easier than FO B cells, which quickly allow them to produce immunoglobulin M (IgM) or induce class switching without T-cell help [58].

The third naïve B cell subset is B-1 cells, which are considered part of the innate immune system [59, 60]. Apart from the other B cell subsets developed in the bone marrow, B-1 B cells originate from a distinct progenitor in the fetal bone marrow [61]. They are mainly found in peritoneal and pleural cavities; however, low numbers can also be located in secondary lymphoid organs. During an infection, they act by producing non-specific antibodies that are crucial for early defense [62, 63].

Advancing age alters the entire course of B cell development, the abundance of distinct B cell subsets, and their function. Furthermore, a B cell subset emerging with increasing age influences immune responses in the elderly.

#### Effects of Aging on B Cell Development

B cell development and the influence of old age in this process are extensively studied in mice. First of all, the differentiation capacity of long-term HSCs (LT-HSCs) reduces with advanced age [64]. The genes driving lymphoid cell differentiation and function are downregulated in LT-HSCs, while the genes mediating myeloid cell development are upregulated. Numbers and percentages of early B-cell lineage progenitors decrease as C57BL/6 mice age [65]. Furthermore, these populations exhibit declined IL-7 responsiveness, indicating an impaired B lymphopoiesis.

Following progenitor differentiation, the development of B cells in the bone marrow is also influenced by aging. In different groups of old mice, a severe decrease with more than 80% loss of pre-B cells and 50% loss of pro-B cells, or a moderate decrease with 20–80% loss of pre-B cells were observed [66]. TFs regulating B cell development are altered by age, influencing the abundance of developing B cells [66–68]. Among them, the *E2A* gene encodes for two proteins, E47 and E12. Transcription and DNA-binding capacity of E47 were shown to decline in aged mice [66]. As E47 is a vital TF in B cell development during the pro- to pre-B cell stage [69], lower numbers of pre- and pro-B cells in old mice could partly be explained by the decreased function and expression of E47. PAX5 is another TF regulating early B-cell development that is lower in the elderly [70]. Lastly, BCR expression and diversity are altered upon aging [71, 72], although a study suggested that the changes were not evident until 70 years of age [73].

#### The Emergence of Age-Associated B Cells

In 2011, a new subset of B cells was described in aged mice [74, 75]. This mature B cell population is named age-associated B cells (ABCs) since it progressively accumulates with increasing age. The origins of ABCs are not exactly known; however, differentiated FO, MZ, and B-1 cells are thought to contribute to the heterogeneous ABC pool [76]. Although studies define ABCs using different markers, they agree that ABCs are mature B cells with memory characteristics. Unlike the other B cell subtypes, ABCs express the transcription factor T-bet and a unique surface marker combination [77]. Therefore, their activation requirements, functions, and survival conditions are remarkably different. BCR engagement induces FO and MZ B cell proliferation, while Toll-like receptor 9 (TLR9) or TLR7 signaling with or without BCR ligation drives proliferation in ABCs [76]. In vitro studies showed that TLR stimulation leads to IL-10 and IFNγ production from ABCs, and an in vivo study reported that they also produce tumor necrosis factor alpha (TNFα) [78].

ABCs are engaged in both protective and autoreactive immune responses, although their protective role seems scarce. Furthermore, they are linked with autoinflammatory and autoimmune diseases, such as systemic lupus erythematosus and rheumatoid arthritis [75, 79, 80], making ABCs a potential underlying reason for the increased incidence of autoimmune diseases in the elderly.

ABCs contribute to immune dysfunctions observed during the aging process. For instance, TNFα produced by ABCs has direct and indirect effects on pro-B cell numbers: ABCs directly induce pro-B cell apoptosis and lead to their loss by altering the bone marrow microenvironment [78]. Besides, increased abundance of ABCs was significantly correlated with the loss of B cell precursors in the bone marrow of aged mice.

ABCs express considerably high major histocompatibility complex II (MHC-II), CD80, and CD86 compared to FO B cells; therefore, they are better inducers of T cell activation and antigen presentation [81]. However, the same study associated these properties of ABCs with autoimmune diseases in an autoimmune-prone mice strain. Besides, considering that they make the bone marrow environment more inflammatory via the production of TNFα and robustly produce IL-6 and IFNγ upon TLR7 and TLR9 engagement [74, 78], it is plausible to propose that ABCs contribute to inflammaging.

Lastly, a study reported that humoral response depends more on TLR signaling and less on CD4^+^ T cell help due to decreased FO B cells and increased ABCs in aged mice [82]. This eventually resulted in impaired production of IgG and long-lived plasma cells.

#### Abundance and Functions of B Cells in the Elderly

Several studies reported a decrease of mature B cell subsets in humans with aging, although the extent of these changes varies depending on the subsets, experimental approaches, and cohorts of people [53, 83, 84]. For instance, Muggen et al. reported that numbers and relative abundance of several B cell subsets including transitional B cells, memory cells, and plasmablasts reduced with aging, particularly in individuals older than 70 years old [73]. Plasma and memory B cell percentages in the circulation and bone marrow decline, while naïve and immature B cells remain relatively stable in older people [85]. The abundance of B-1 cells, along with their ability to produce IgM, decreases with age [63]. A study found significantly low switched memory B cells, but high naïve and double-negative memory B cells in people over 65 years of age compared to younger adults [86]. The authors concluded that double-negative or so-called late-exhausted memory B cells express senescence markers and are associated with poor immune responses against influenza vaccine. Of note, switched memory B cells play a role in antibody production upon re-infection, generating a rapid response compared to naïve B cells [84]; therefore, a lower abundance of switched memory B cells is another evidence of impaired humoral immune response in the elderly.

Not only the numbers but also functions of B cells are diminished with aging. Poor antibody responses in the elderly after influenza vaccination are due to low binding and neutralization capacity of antibodies, decreased class switch recombination, hypermutations of the antibody variable regions, and higher abundance of inflammatory B cells [87, 88]. Besides, antigen-specific antibody production decreases with age, while self-reactive antibodies become more abundant, rendering old individuals more susceptible to develop autoimmune diseases [89]. All these defects in the humoral immune response lead to increased susceptibility to diseases and reduced efficiency of vaccines [90].

## Trained Immunity: a De facto Innate Immune Memory

Although immune memory had been attributed only to the adaptive immune system for a long time, growing evidence consistently shows the existence of memory-like characteristics in innate immune cells [91–94]. Certain infections, vaccinations, or molecules can reprogram innate immune cell types to exhibit increased responsiveness against a secondary insult. This phenomenon is termed trained immunity and mediated through extensive epigenetic and metabolic changes.

Over the last couple of years, innate immune cells, including monocytes [95], natural killer (NK) cells [96], innate lymphoid cells (ILCs) [97], DCs [98], and neutrophils [99], have been reported to exhibit trained immunity response. As innate immune cells can only recognize microbial patterns via their pattern recognition receptors (PRRs), their memory-like response is not specific to pathogens but can work against a wide range of antigens. Thus far, vaccines, such as the tuberculosis vaccine Bacillus-Calmette Guérin (BCG) [100], measles [101], and oral polio vaccine [102]; microbes/microbial patterns, e.g., β-glucan [91], *Candida albicans*; oxidized low-density lipoprotein (oxLDL) [103]; and metabolites such as fumarate [104] have been reported to induce heterologous protection through trained immunity.

Epidemiological studies reporting decreased all-cause mortality after certain vaccinations suggested the existence of an innate immune memory [105]. The existence of trained immunity was first depicted in monocytes with an in vitro model and in vivo in mice, where *C. albicans* and β-glucan induced enhanced cytokine productions after the second microbial stimulation [91]. In parallel, BCG vaccination was reported to induce higher TNFα and IL-1β production against unrelated pathogens, even 3 months after the vaccination [100]. Further research demonstrated that trained immunity could persist up to 1 year and possibly even longer [106]. Considering that monocytes have a half-life around 1–2 days in the circulation [107], the programming of progenitor cells could be involved in sustaining the memory-like phenotype. Indeed, β-glucan administration leads to the expansion of myeloid lineage progenitors in the bone marrow of mice [108]. Increased myelopoiesis is associated with upregulated IL-1β and granulocyte–macrophage colony-stimulating factor (GM-CSF) signaling, besides alterations in glucose and cholesterol metabolism. Another mouse study demonstrated increased myelopoiesis following BCG vaccination, which is associated with enhanced protection against *M. tuberculosis* infection [109]. These findings align with a recent study on humans, showing that BCG vaccination leads to the upregulation of myeloid and granulocyte-lineage genes in HSCs [110].

### Trained Immunity in the Elderly

Low-grade chronic inflammation occurring in the elderly is associated with poor innate and adaptive immune responses [111]. Koeken et al. recently reported that BCG vaccination reduces systemic inflammation, and a lower abundance of circulating inflammatory proteins at baseline is correlated with trained immunity response 3 months after vaccination in males [112]. Therefore, BCG vaccination could alleviate inflammaging while providing non-specific protection via trained immunity induction in the elderly. On the other hand, since the cell differentiation capacity of HSCs in the bone marrow changes and is skewed toward myelopoiesis with aging, inducing trained immunity could lead to unfavorable outcomes by further expanding the myeloid cell production in older people.

Nevertheless, a double-blinded placebo-controlled clinical trial demonstrated that trained immunity could be safely induced in the elderly by BCG vaccination, evident from the increased cytokine production compared to the participants who received placebo [113]. Remarkably, the trial showed that BCG prolongs the time until an infection and reduces the risk of all new infections and respiratory infections by 45% and 79% compared to the placebo group, respectively. In line with this, other trials reported a decrease in acute upper respiratory tract infections and pneumonia in older people vaccinated with BCG [114, 115]. However, more research is needed to explore the strength and longevity of trained immunity responses in older individuals compared to adults.

BCG’s ability to confer protection against heterologous infections has attracted a lot of attention during the COVID-19 pandemic, which disproportionally affects the elderly. BCG is being tested in more than 20 randomized control trials to investigate if it has a protective effect against SARS-CoV-2 infection [116]. Promisingly, a recently published study from Greece reported 68% risk reduction for COVID-19 6 months after BCG vaccination [117]. Another study revealed that even an early history of BCG vaccination is associated with decreased incidence and symptoms of COVID-19 among healthcare workers [118]. Therefore, induction of trained immunity by BCG vaccination may be utilized as a preventive measure against COVID-19, especially in the vulnerable elderly group.

## Aging as a Multisystem Malady

Aging leaves no part of the body unscathed. Besides tissue-specific damage occurring with advanced age, the aging immune system impacts many other systems and processes. Even the organs that were once thought to be devoid of immune cells, such as the brain, are now known to harbor tissue-resident immune cells and interact extensively with the peripheral immune system. The last few decades have also witnessed a boom in research on the microbiota, the collection of up to 100 trillion microorganisms residing in human bodies, mainly in the gut [119]. The microbiota has close interactions with the host immune system and is also prone to age-related disruptions.

**Fig. 1:**
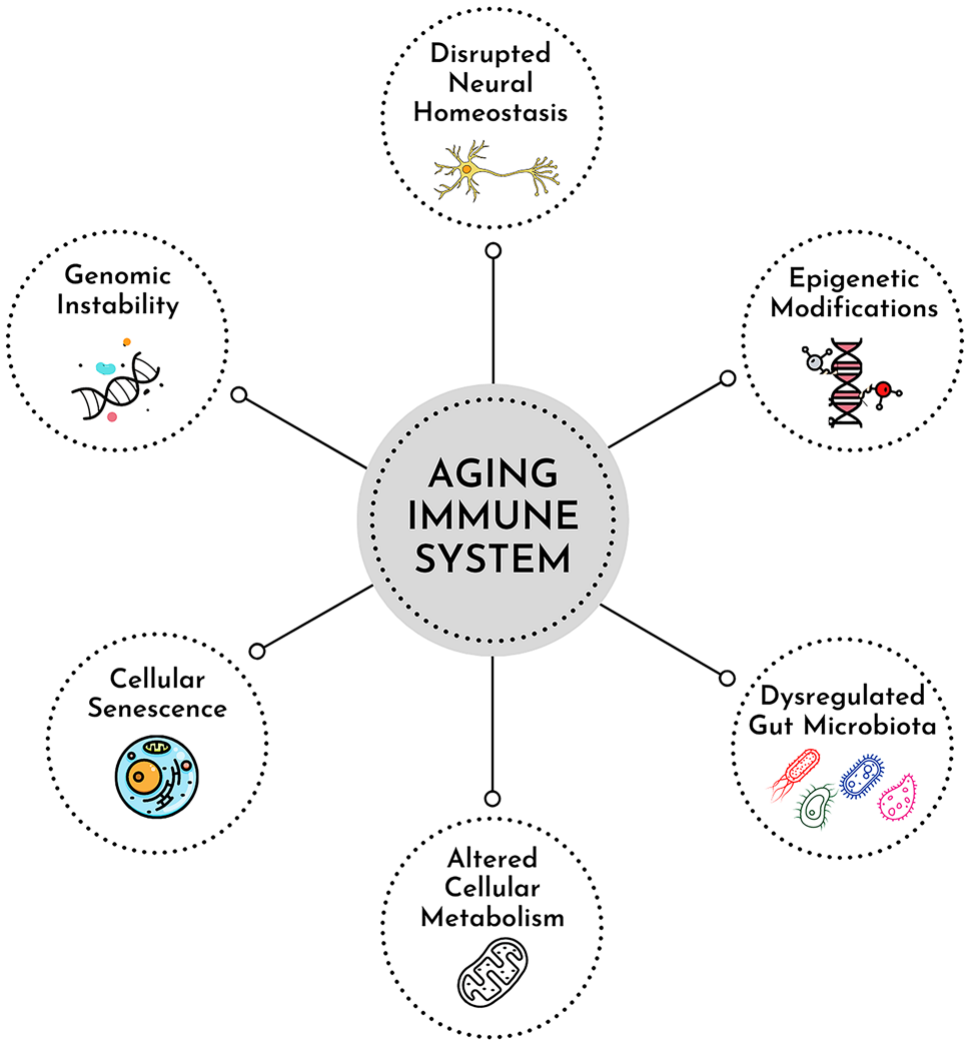
The far-reaching effects of the aging immune system. Age-related changes in immune cells include genomic instability, epigenetic modifications, altered cellular metabolism, and cellular senescence. An aged and impaired immune system has broad consequences, affecting many tissues and systems of the body. Gut microbiota and the central nervous system are profoundly impacted by and, in turn, regulate the immune system

In the following chapters, we discuss the interplay of microbiota and the brain with the aging immune system, mainly focusing on immune memory. We especially approach this body of research from a metabolic perspective, describing various cellular metabolic programs and their impact on immune memory in aging and age-related diseases. Additionally, we point out the role of epigenetic regulation underlying all the topics discussed. By providing such a comprehensive view, visualized in Fig. 1, we aim to strengthen the notion of aging as a multisystem problem and accordingly inform counteractive efforts.

### Interplay of Metabolism and Immune Memory

Metabolism and metabolic inflammation are key processes that both influence and get influenced by aging. Metabolic diseases such as type 2 diabetes mellitus, cardiovascular diseases, and obesity are also considered age-related diseases. These conditions are accompanied by chronic inflammation, termed metaflammation, which is driven by nutrient excess. Although the triggers might vary, the mechanisms underlying metaflammation and inflammaging are quite similar. Mitochondrial dysfunction, accumulation of senescent cells and cellular debris, and hyperactivation of innate immune responses, such as inflammasome, contribute to both processes [120]. Therefore, it is crucial to understand the interplay between cellular aging, metabolism, and inflammation in chronological aging and age-related metabolic diseases to revert them.

#### T Cell Metabolism

Quiescent T cells mainly use catabolic processes, while activated cells rely on anabolic processes to support protein production and proliferation. Cells need to activate a critical serine/threonine kinase, mammalian target of rapamycin (mTOR), to induce anabolic pathways [121]. While driving growth and proliferation, mTOR also upregulates glucose transport and glycolysis. Glycolysis is one of the main pathways to generate energy. Although it is not energetically efficient — only 2 adenosine triphosphate (ATP) molecules can be generated from one glucose molecule — it generates energy very rapidly, which is of use for active and proliferating T cells [122]. Processing of glucose yields ATP, NADH, and pyruvate. Pyruvate is then converted to lactate and exported as lactic acid in the case of glycolysis or otherwise transported to mitochondria for oxidative phosphorylation (OXPHOS).

OXPHOS is a much more efficient bioenergetic pathway, producing 36 ATP molecules from every glucose molecule [123]. In this case, pyruvate is converted to acetyl-CoA and enters the tricarboxylic acid cycle (TCA cycle), which is coupled to the electron transport chain (TCA) through electron donors NADH and FADH2. TCA cycle can be replenished by amino acids and oxidation of fatty acids. Fatty acid oxidation (FAO) is mainly used by cells with low energy demands and plays a critical role in CD8^+^ memory and CD4^+^ Treg development [124]. Activated T cells upregulate their glutamine uptake and perform glutaminolysis to yield α-ketoglutarate, which enters the TCA cycle.

Additionally, TCA cycle metabolites can regulate immune functions in ways other than energy production. For instance, acetyl-CoA acts as the key cofactor for histone acetylation [125]. In activated T cells, acetyl-CoA is required for IFNγ production through histone acetylation [126]. Acetyl-CoA also contributes to the acetylation of mitochondrial proteins [127], which has vast functional consequences for both innate and adaptive immune cells [128].

Quiescent naïve T cells meet their energy needs with OXPHOS [129]. IL-7 and TCR signaling are essential for their metabolic regulation and survival [130, 131]. When T cells are activated, an immediate need for energy occurs for effector functions and biomass generation. The cells upregulate transporters like glucose transporter 1 (GLUT1) and engage in aerobic glycolysis, promoting cytokine production through pathways, such as the phosphoinositide 3-kinase (PI3K)-AKT-mTOR axis and mitogen-activated protein kinase (MAPK) signaling [132]. The glycolytic switch is required for the effector functions, e.g., IFNγ production but not essential for proliferation [133]. OXPHOS can also be utilized for proliferation and survival purposes. Although activated T cells functionally rely on glycolysis, OXPHOS is certainly not dispensable: when OXPHOS is inhibited with oligomycin, T cell activation and proliferation are blocked [133].

Although they rely on OXPHOS and FAO in the resting state, memory T cells need to respond quickly and efficiently upon antigen encounter. Therefore, they can shift to glycolysis quicker than naïve T cells [134]. Greater mitochondrial mass and a strong mitochondrial spare respiratory capacity have been linked to this bioenergetic advantage [135, 136]. Additionally, mitochondrial fusion is essential for the development and function of memory T cells [137].

#### Impact of Aging on T Cell Metabolism

Increased p38 MAPK activity is one of the characteristics of senescent T cells. Inhibiting p38 improves telomerase activity, proliferation, autophagy, and mitochondrial fitness, in an mTOR-independent way [17]. MAPK inhibition also enhances T cell and antibody responses in influenza-vaccinated old mice [138].

Patients with gain-of-function mutations in PI3K have depleted naïve T cells but an accumulation of senescent effector cells, just like in the elderly [139]. Inhibiting mTOR activity with rapamycin treatment partially restores the senescent phenotype in these patients. Therefore, overactive PI3K/AKT/mTOR signaling is suggested as one of the drivers of T cell senescence.

Aged naïve T cells have higher mitochondrial mass, but interestingly, less mitochondrial respiratory capacity, possibly due to transcriptional downregulation of respiratory chain genes [140]. Furthermore, enzymes of one-carbon metabolism are deficient in aged naïve T cells, and supplementation with formate and glycine, one-carbon metabolism metabolites, improves cell survival and activation [141].

Autophagy is important for the generation of T cell memory, and induction of autophagy by spermidine improves CD8^+^ T cell responses against influenza vaccination in aged mice [142]. CD4^+^ memory T cells of the elderly display upregulated oxidative phosphorylation, reactive oxygen species (ROS) production, and fatty acid oxidation [143]. They also have a higher expression of Sirtuin 1 (*SIRT1*), a NAD-dependent deacetylase, compared to younger cells. SIRT1 and AMPK, two important nutrient-sensing molecules and negative regulators of mTOR, positively influence each other [144]. In contrast to CD4^+^ memory cells, aging-associated terminally differentiated memory CD8^+^CD28^−^ T cells have a high glycolytic capacity, which is linked to their downregulated *SIRT1* expression [145].

CD8^+^ TEMRA cells have a higher expression of glycolysis and glutaminolysis-related genes and a larger ATP pool compared to naïve and EM cells [146]. Despite upregulated glycolytic transcription in TEMRA cells, basal glycolysis levels are similar to naïve and EM cells. Like EM cells, TEMRA cells can quickly increase glycolysis and OXPHOS upon activation [146]. In terms of function, TEMRA cells are capable of cytotoxicity and cytokine production, despite their senescent state and impaired mitochondrial function [17, 36].

Long-term CMV infection, known to promote immunosenescence, also alters the cellular metabolism of T cells, increasing glucose uptake, promoting glycolysis, restructuring lipid rafts, and disturbing cholesterol metabolism [147, 148]. In addition, chronic inflammation due to lifelong CMV infection disrupts pancreatic β-cells and increases the risk for type 2 diabetes in the elderly [149].

#### B Cell Metabolism

The metabolic pathways that regulate T cells are also essential for B cell function, although there has not been much research on B cell metabolism. When a B cell is activated upon antigen recognition by the BCR and T cell help, it activates PI3K/AKT/mTOR signaling [150]. Just like activated T cells, activated B cells need rapid energy production to increase biomass and proliferate. As a result, glucose and glutamine uptake increase, along with oxygen consumption, OXPHOS, and mitochondrial remodeling [151]. OXPHOS and glutamine-fueling of the TCA cycle have been suggested as the critical bioenergetic pathways for B cell growth and function, while glucose was dispensable [152].

A study showed that activated B cells have more mitochondria but similar amounts of mitochondrial DNA, indicating that fission of naïve B cell mitochondria with multiple nucleoids, rather than mitochondrial replication, occurs upon activation [152]. Another study suggested that mitochondrial remodeling and ROS levels determine the fate of activated B cells. Cells with increased mitochondrial mass and higher ROS levels upon activation are destined for class switch recombination, whereas cells with decreased mitochondrial mass undergo plasma cell differentiation [153].

The energy needs of activated B cells in GCs frequently shift [154]. In the hypoxic light zone, cells consume less oxygen and are more glycolytic. mTORC1 is not necessary for the regulation of glycolysis here, but it is critical, together with c-Myc, for the positive selection of the cells and migration to the dark zone for proliferation and somatic hypermutation [155, 156].

Upon GC maturation, when a cell differentiates into memory B cell, the metabolic state becomes more quiescent with dominant OXPHOS. However, rapid re-activation of mTORC1 and glycolysis is possible for later differentiation into antibody-producing plasmablasts [157]. Furthermore, memory B cells have high basal autophagy, which is essential for their survival until antigen encounter [158, 159].

GCs also output long-lasting plasma cells, which can produce thousands of antibodies per second. This, naturally, is highly energy demanding. mTORC1 is essential for plasma cell generation and antibody synthesis [160]. Plasma cells have high levels of glucose uptake, but most of the glucose is used for protein glycosylation [161]. Still, survival and antibody production of plasma cells were impaired when the glucose transporter Glut1 was deleted [162]. Also, mitochondrial import of pyruvate, provided by glycolysis, is critical for the long-term maintenance of plasma cells [161].

Finally, tissue-resident B1 B cells are more active in glycolysis and OXPHOS than other B cells, the classical antibody-producing and memory B cells. In addition, autophagy is critical for the mitochondrial function and self-renewal of B1 cells [163].

#### Impact of Aging on B Cell Metabolism

There is less literature on how B cell metabolism is regulated and impacts function as organisms age. A study showed that antibody-secreting B cells of aged individuals had lower *SIRT1* expression, and higher *SIRT1* levels were associated with better antibody response to multiple influenza virus strains [164]. Also, naïve and activated B cells of the elderly had slightly less glycolytic capacity and a more striking reduction in OXPHOS. In mice, aged B cells had similar glycolysis and OXPHOS rates as young counterparts but could not further enhance OXPHOS upon stimulation [165]. However, the cells were able to upregulate glycolysis to meet their energy need.

Leptin, a pro-inflammatory hormone secreted by adipocytes, is higher in the circulation of obese individuals [166]. Among non-obese people, leptin concentrations are strikingly more elevated in the elderly [167]. Leptin abundance in the serum is also positively associated with frailty [168]. After exposure to leptin, B cells from young lean individuals exhibit a similar profile as B cells of older lean and young obese individuals regarding the transcriptional profile and antibody secretion [167]. Leptin also decreases influenza-specific antibody production from B cells in vitro. Obesity is known to impair B cell responses to vaccination, and studies suggest that leptin might be partially responsible for this [169].

Additionally, post-transcriptional glycosylation of antibodies modulates their function, and altered glycosylation patterns have been linked to aging [170, 171]. β4-Galactosyltransferase activity increases with age [172], which would have functional consequences, although yet unexplored.

#### Metabolism in Trained Immunity

Metabolic reprogramming is one of the key mechanisms underlying trained immunity (also known as innate immune memory), along with chromatin remodeling. In fact, metabolic changes can drive epigenetic changes since certain metabolites, e.g., acetyl-CoA, can regulate epigenetic enzymes [173]. Fumarate is one example of TCA metabolites driving epigenetic changes. It can induce trained immunity on its own, and its accumulation during this process induces trimethylation of histone 3 lysine 4 at the promoters of IL-6 and TNFα [104]. This is due to fumarate inhibiting the activity of lysine-specific histone demethylase KDM5.

The AKT/mTOR/HIF1α pathway is the most critical pathway for inducing aerobic glycolysis in β-glucan-trained monocytes [174]. Contrary to β-glucan-induced trained immunity, BCG upregulates not just glycolysis but also OXPHOS [175]. Glutaminolysis and cholesterol synthesis are other crucial metabolic pathways for β-glucan-induced trained immunity [104]. Interrupting these pathways blocks these processes in vitro and in vivo. BCG also induces glutaminolysis, and glutamine availability is important for the trained response [175].

Synthesis of cholesterol itself is not essential for trained immunity but rather the accumulation of the intermediate mevalonate is. Blocking mevalonate generation inhibits trained immunity, while mevalonate alone can induce trained immunity in monocytes through the activation of insulin-like growth factor 1 (IGF1) receptor and mTOR [176]. Furthermore, the changes in glycolysis and mevalonate pathways are observed not only in monocytes but also in HSPCs [108].

oxLDL, a non-microbial inducer of innate immune memory, upregulates both glycolysis and oxygen consumption, and high glucose availability further enhances the trained immunity response [103]. Similarly, catecholamine-induced trained immunity is accompanied by increased glycolysis and oxygen consumption. Of note, the particular metabolic rewiring might differ for different inducers of innate immune memory. For instance, stimulation with aldosterone is not associated with elevated glycolysis or OXPHOS but is dependent on fatty acid synthesis [177].

As of yet, trained immunity responses and associated metabolic states have not been characterized in the context of aging. However, several ongoing large-scale studies of BCG vaccination in the elderly would soon shed light on the effects of BCG-induced trained immunity on the metabolism of aged immune cells (NCT04537663, NCT04417335).

### Role of Epigenetic Alterations in Immune Memory

Epigenetic changes include histone modifications and DNA methylation that regulate the way a gene works. These modifications are dynamic and affect all cells and tissues throughout life. Environment and lifestyle, as well as aging, can lead to dramatic epigenetic alterations. For the purpose of this review, we will focus on how age-dependent epigenetic modifications alter innate and adaptive immune memory.

#### DNA Methylation In Adaptive Immunity

DNA methylation is the most abundant epigenetic modification that occurs by transferring a methyl group to the 5th carbon of the cytosine [178]. DNA methylation does not always indicate a lower gene expression; however, methylation in gene promoters is generally associated with poor TF binding and reduced transcription [179]. Biological sex, genetic background, environmental factors, and age affect the DNA methylation profile [180]. Among these factors, age-dependent methylation is very well-characterized. Remarkably, different mathematical models are developed to predict the biological age based on the methylation levels of certain CpG sites from various tissues or cells [180–182].

Advancing age is associated with a progressive loss of methylation marks on DNA [183], although abnormal hypermethylation patterns are also observed in some gene promoters [184]. Changes in the methylation landscape are linked to various age-related diseases as well as dysfunctions in the immune system. For instance, age-related macular degeneration, a disease resulting in irreversible blindness in the elderly, has been correlated with the loss of methylation in the promoter region of the *IL17RC* gene, leading to increased IL17RC protein levels in the blood [185].

A growing number of studies indicate that DNA methylation plays a significant role in the adaptive immune system’s functioning. Age-related functional changes in immune cells, such as decreased self-renewal capacity, defects in cell differentiation, and skewed differentiation towards myeloid cell production in the elderly, are strongly correlated with epigenetic modifications occurring in HSCs during aging [186]. Murine studies show that gene expression of HSCs is regulated via hyper- and hypomethylation of certain DNA regions, affecting the capacity of those cells to differentiate [187]. Expression of DNA methyltransferases in HSCs are lower in aged mice [186]. Also, HSCs of mice with decreased DNA methyltransferase activity fail to efficiently differentiate into lymphoid progeny [188]. These studies reveal that DNA methylation is essential to fine-tuning the differentiation capacity of HSCs and therefore the proper activity of the innate and adaptive immune system. Epigenetic modifications also modulate the function of HSCs during aging, which will be elaborated in “Histone Modifications.”

Several studies report age-dependent methylation changes in T cells. A study analyzing CD4^+^ and CD8^+^ T cell methylome profiles in young individuals and the elderly found that 48,876 and 12,275 CpG sites were differentially methylated in CD8^+^ T and CD4^+^ T cells, respectively [189]. Moreover, the methylation profile of CD8^+^ T cells was strongly associated with aging and inversely correlated with genes linked to T cell differentiation and immune response, suggesting a possible link between weakened T cell responses and age-related alterations in DNA methylation.

The age-associated methylation profile of CD4^+^ T cells is characterized by hypermethylation of CpG sites enriched in the polycomb repressive complex 2 (PRC2) genes and hypomethylation of CpG sites enriched in enhancer regions [190, 191]. Of note, the PRC2 proteins regulate histone methylation, cell differentiation, and proliferation [192]. These methylation patterns identified by Dozmorov and colleagues were highly similar to the methylation and transcriptomic profile of T cells from lupus patients. Lupus erythematosus, an autoimmune disease leading to autoreactive T cells, is characterized by defects in the MAPK signaling pathway and increased mTOR activity resulting from altered methylation patterns [193]. Therefore, the authors suggested that the age-dependent methylation profile of naïve CD4^+^ T cells might render the elderly susceptible to autoimmune diseases, such as lupus, though this remains to be formally demonstrated.

Loss of CD28 co-stimulatory protein in CD4^+^ T cells is one of the well-characterized aging marks, leading to impaired T cell activation and differentiation. Comparison of methylation profiles of CD28^+^ and CD28^null^ T cells revealed 296 differentially methylated genes associated with poor TCR signaling and cytotoxic response [194]. Furthermore, the expression of the genes involved in inflammasome activation was higher in CD28^null^ T cells, suggesting that these cells have a higher pre-activation state. Another study reported that increased methylation at the BACH2 locus of the CD4^+^ T cells in the middle and old age groups results in lower *BACH2* expression [195]. BACH2 has a regulatory role in immune responses, modulating CD4^+^ T cell differentiation and controlling inflammation [196]. Overall, alterations in the DNA methylation patterns contribute to CD4^+^ T cells becoming more inflammatory in the elderly.

A few studies shed light on the DNA methylation profile of B cells during activation and diseases [197–200]; however, whether B cells are affected by age-dependent methylation changes is yet to be known.

#### Histone Modifications in Adaptive Immunity

N-terminal histone tails are targets for post-translational enzymatic modifications including acetylation, methylation, phosphorylation, ubiquitylation, and sumoylation [201]; however, this review will focus on methylation and acetylation, which are the most well-characterized alterations regulating histone structure. Methyl groups are added to the histone by histone methyltransferases and removed by histone demethylases [202]. The trimethylation of histone 3 lysine 4 (H3K4me3), histone 3 lysine 36 (H3K36), and histone 3 lysine 79 (H3K79) are linked to open and actively transcribed regions [203]. On the other hand, mono-methylation of histone 3 lysine 9 (H3K9me), histone 3 lysine 27 (H3K27me), and histone 4 lysine 20 (H4K20me) is associated with closed and inactive chromatin regions. Furthermore, histone acetylation is associated with loosened chromatin structure and increased gene transcription [204]. Histone acetyltransferases catalyze lysine acetylation, whereas histone deacetylases (HDACs) reverse the modification [205]. Post-translational modifications of histones do not only influence the accessibility and transcription of genes but also modulate alternative splicing, DNA replication, and repair [206].

Histones and epigenetic marks on histones undergo transitions with aging. HSCs from old mice have more H3K4me3 and H3K27me3 peaks compared to young HSCs [186]. In addition*,* expression of *FLT3*, one of the regulators of CLPs, was decreased due to H3K27me3 in the old HSCs, suggesting a link between poor lymphoid differentiation potential of HSCs in the elderly. An extensive study performed in young and old monozygotic twins showed that chromatin modifications during aging are non-heritable [207]. Moreover, histone modification profiles are, to some extent, homogenous in young individuals and heterogeneous among elderly subjects. Heterogeneity in histone modifications was observed between individuals and also cell types in the elderly.

Epigenetic changes are one of the underlying causes of the major defects seen in CD8^+^ T cells of the elderly. More closed chromatin regions are observed in the enhancer and promoter regions of the genes related to T cells signaling in the elderly compared to the young [208]. Furthermore, *IL-7R*, in the memory CD8^+^ T cells, is one of the top genes related to multiple closed chromatin peaks in the elderly. As IL-7 ensures homeostasis and maintenance of T and B cells, poor IL-7 signaling in the elderly might be an underlying reason of impaired adaptive immune response [209]. Furthermore, naïve CD8^+^ cells in the elderly have lower chromatin accessibility at the gene promoters associated with poor nuclear respiratory factor 1 (NRF1) binding [140]. Considering the role of NRF1 in oxidative phosphorylation, decreased chromatin activity might partially explain the impaired CD8^+^ T cell metabolism in the elderly [210]. Other significant findings of the study are that open chromatin regions are associated with a memory cell profile, and accessibility of the promoters is diminished in aged individuals.

As mentioned in the DNA methylation section, an age-associated decrease in *BACH2* expression is observed in CD4^+^ T cells. Another mechanism leading to lower *BACH2* gene transcription is due to Menin deficiency observed in immune senescence [211]. Menin induces *BACH2* expression by binding to its locus and maintaining histone acetylation. Decreased binding of Menin to *BACH2* locus and subsequently reduced *BACH2* expression contributes to immunosenescence in CD4^+^ T cells.

A study investigating the epigenetic changes in B cell precursors in old and young mice associated these alterations with gene expressions [212]. It revealed that aged pre-B cells exhibit a loss of H3K4me3 at the promoter site of insulin receptor substrate 1 (*IRS1*), which is associated with lower transcription. As insulin signaling is necessary for the development of B cells in the bone marrow [213], decreased insulin growth factor (IGF) signaling might lead to defects in B cell development.

#### Epigenetic Reprogramming as a Hallmark of Trained Immunity

A distinct epigenetic profile regulates trained immunity responses following the first insult. As a result of certain infections or stimulations, primed cells undergo an epigenetic reprogramming that allows them to respond stronger upon a heterologous infection by facilitating the transcription of genes related to inflammation and metabolism [106].

H3K4me3 is the first characterized epigenetic mark in monocytes after β-glucan treatment [91]. Further analysis revealed that H3K4me3 peaks are enriched at the promoter sites of *TNF*, *IL6*, *IL18*, *DECTIN1*, and *MYD88* genes, indicating that gene transcriptions are more active in these regions. In addition, increased H3K27ac is a well-characterized histone mark in trained cells, promoting glycolysis and PI3K/AKT pathway activation [174, 214]. Besides the enrichment in H3K4me3 and H3K27ac, decreased H3K9me3 was found in the promoters of genes related to cytokine production and glycolysis [175]. Since H3K9me3 is a repressive mark, reduced trimethylation suggests the presence of open chromatin regions. These studies show that trained immunity responses are modulated by epigenetic modifications that facilitate enhanced cytokine responses and specific metabolic changes. Trained cells share a common epigenetic profile; however, different stimuli could lead to minor unique epigenetic alterations.

Infections and certain stimulations leave marks on the DNA methylation profile, as well as histones, of innate immune cells [215]. Studies demonstrate the role of DNA methylation in anti-mycobacterium response following BCG vaccination, discriminating responders from non-responders [216, 217]. Responders to BCG vaccination were characterized by reduced DNA methylation at the promoters of inflammatory genes [216]. However, whether DNA (de)methylation plays a direct role in the development of non-specific protective responses is still being investigated.

As in adults, trained immunity is modulated by histone modifications in the elderly. Giamarellos-Bourboulis and colleagues recently showed that increased cytokine production upon BCG vaccination in the elderly was accompanied by acetylation of H3K27 at the promoter regions of *TNF* and *IL6* genes [113]. However, further studies are warranted to compare the epigenetic differences following innate immune memory development between adults and older individuals and explore how aging influences epigenetic marks in the context of trained immunity.

### Gut Microbiota Modulating Immune Memory

Aging causes changes throughout the whole body of humans, and trillions of microbes living there are no exemption. The composition and diversity of gut microbiota dynamically shift in infancy, remain relatively stable during adulthood, and start to decline with old age [218].

#### Interactions of Microbiota and the Adaptive Immune System

The gut microbiota has essential roles in educating the adaptive immune system by inducing a certain level of immune response and fine-tuning the inflammation. For instance, *Bacteroides fragilis*, a commensal in the gut, enhances and regulates CD4^+^ T cell differentiation into T helper 1 (Th1) and Th2 [219]. In the presence of gut bacteria and TGFβ, naïve CD4^+^ T cells become Tregs, producing IL-10 to maintain immune homeostasis. On the other hand, Tregs and Th17 cells in the lymphoid follicles of the gut induce B cell class switching, resulting in IgA secretion [220, 221]. Microbiota-associated IgA, IgM, and IgG secretion from B cells also occurs via TLR signaling activation without T cell help [222].

The adaptive immune system can limit the inflammatory response against commensal gut microbes mediated by the innate immune system. IgA produced by B cells is explained as a part of sustainable host-microbe interaction, controlling the inflammatory response against beneficial microorganisms [223]. Besides, intestinal Treg cells express TCRs for intestinal antigens, such as metabolic products and commensals, while other Tregs in the body express TCRs for self-antigens [224]. In this way, intestinal Tregs suppress immune responses against intestinal antigens and play an immunoregulatory role in the guts.

How microbiota strikingly shapes the adaptive immune system development was also demonstrated in germ-free mice: the lack of microbial species in the gut is characterized by defects in secondary lymphoid tissue development [225], low IgA production [226], and reduced Th17 cells and Tregs [227]. It should be noted that short-chain fatty acids (SCFAs) produced by microbial species in the gut greatly contribute to the immune system development and responses [228].

A healthy gut microbiota composition is important in protecting individuals from diseases. As an example, IL-10 secreting IgA + plasma cells and plasmablasts originating in the gut confer resistance to experimental autoimmune encephalomyelitis induced in mice [229]. Another study reported that gut microbiota protects against respiratory infections induced by *S. pneumoniae* and *K. pneumoniae* by inducing GM-CSF and IL-17A secretion [230].

#### The Role of Dysbiosis in Aging

The incidence of gut dysbiosis, the imbalance of microbial species, increases with age and is associated with numerous health problems [231]. However, it is unclear whether cellular and molecular alterations of the immune cells during aging affect the composition and functioning of the gut microbiota, or if age-related dysbiosis triggers defective immune responses. It is likely that both are concurrently true, but a better understanding of the gut microbiota-immune system interactions is necessary to resolve this question.

As individuals age, a decline in certain beneficial bacterial species, such as *Bifidobacterium*, is replaced by the growth of pathogenic species, i.e., *Enterobacteriaceae* [232]. A decrease in *Firmicutes* and increase in *Proteobacteria* are also reported in older people [233]. Besides, gut dysbiosis is associated with several age-related diseases, including obesity [234], type 2 diabetes [235], Alzheimer’s disease [236], and increased incidence of infections [237–239]. The risk of developing cancer is also higher in the elderly due to dysbiosis-associated chronic inflammation, debilitated phagocytosis of senescent and dormant tumor cells, and impaired activation of tumor-specific CD8^+^ T cells [240].

Dysbiosis was also proposed to be a major reason for various age-associated pathologies and premature death in older individuals by triggering excess inflammation and several complications, including leaky gut and diminished functions of gastrointestinal tract [228]. In line with this, a particular composition and diversity of microbial species is correlated with health, fitness, and increased survival in the elderly [241, 242]. A recent study revealed that healthy elderly experience a particular drift in their microbiota composition, while this drift is missing in the frail elderly [242]. Furthermore, having high *Bacteroides* abundance during aging correlates with decreased survival rate over the 4-year follow-up. Another recent work with 15 years of follow-up reported that *Enterobacteriaceae* abundance was significantly linked with deaths related to gastrointestinal and respiratory causes in the elderly [243].

Dysbiosis can lead to defects in intestinal barrier integrity, which results in the translocation of bacterial species to the host tissues. Those bacteria create inflammation through the recruitment of neutrophils and differentiated Th17 cells [244]. For example, translocation of a gram-positive pathobiont *E. gallinarum* that results from defects in the gut barrier induces Th17 response and autoantibody production [245].

*Akkermansia* is a beneficial commensal shown to protect the gut barrier integrity [228] and enhance antibody and T cell responses [246]. Loss of *Akkermansia* is associated with insulin resistance in aged non-human primates and mice [247]. Decreased butyrate and *Akkermansia* abundance increase gut leakage, which in turn increases pro-inflammatory responses.

A human study, on the other hand, reported that *Akkermansia* is more abundant in the elderly [248]. Furthermore, *Akkermansia* was significantly correlated with serum IgA and CD8^+^ T cells and negatively correlated with CD4^+^ T cells in older people. *Bacteroidetes*, which are less abundant in the elderly, were positively correlated with serum IgG levels and CD4^+^ T cell abundance in the middle age group. In conclusion, this study highlights the relationship between the adaptive immune system and gut microbiota composition, although the direct link between them is missing.

Microbiota also affects disease course and vaccine responses in the elderly. Even though the antiviral therapy for human immunodeficiency virus (HIV) is successful and increases the life expectancy of patients, older HIV + people suffer more from comorbidities compared to HIV − elderly. HIV + elderly have less CD4^+^ T cells and more CD8^+^ T cells than HIV individuals older than 55 [249]. In addition, the abundance of *Prevotella* in the gut is significantly higher in the individuals with low CD4^+^ T cell counts. *Prevotella* was previously associated with cardiovascular diseases [250], but how it interacts with the immune system is not yet clear.

Age-dependent alterations in gut microbiota are likely to contribute to poor immune responses after vaccinations [251]. Some studies reported that probiotics supplements increase the antibody titers after influenza vaccine in the elderly [252–255], whereas a few studies showed limited or no effect [87, 256, 257]. Variations in the results could be due to multiple factors, including the sample size, type of probiotics, and delivery route. Nevertheless, studies strongly suggest that imbalances in microbiota cause impaired immune responses, and restoring the healthy composition might be beneficial for a better vaccine response in the elderly.

#### Innate Immune Memory Induction by Gut Microbiota

As the adaptive immune cells, members of the innate immune system closely interact with the gut microbiota. A few studies suggest that microbiota could regulate immune memory development by priming or tolerizing the cells with microbial antigens and SCFAs. For instance, β-glucan, a fungal cell wall component, and BCG act through Dectin-1 and NOD2 signaling pathways, respectively [91, 100]. Since Dectin-1 and Nod-like receptors (NLRs) are found on various cell types in the intestines, including non-immune cells, it is plausible to propose that these cells develop immune memory due to their exposure to the gut microbiome. Supporting this argument, peptidoglycan fragments derived from gut microbiota were shown to prime the innate immune system, promoting the killing capacity of neutrophils [258].

Furthermore, gut microbiota was shown to induce myelopoiesis to protect mice against infection [259], similar to the increase in the number of myeloid progenitors in the bone marrow of mice following trained immunity induction by β-glucan administration [108]. Other microbiota-derived components, such as lipopolysaccharide (LPS), flagellin, and β-glucan, might also be able to induce trained immunity in the guts, although the dose of the stimuli is critical for immune memory or tolerance response [260].

As mentioned before, trained immunity is mediated by extensive metabolic and epigenetic programming. Molecules and metabolites produced by commensal gut microbes and microbes themselves are able to induce such changes in both innate and adaptive immune cells [261]. For example, despite causing an increase in the anti-microbial activity, butyrate produced by gut microbes have effects opposite to trained immunity in macrophages, possibly stemming from decreased mTOR activity and inhibition of HDAC3 [262].

It is important to note that non-immune cells, e.g., fibroblasts [263], epithelial cells [264], and intestinal stromal cells (ISCs) [265] are also capable of forming immune memory, showing increased responsiveness after secondary infection. It was shown that ISCs could clear infection more rapidly during a secondary related or unrelated infection, indicating the presence of immune memory [266]. Therefore, non-immune cells also contribute to the homeostasis between gut microbes and the immune system.

Considering the strong links between gut microbiota and induction of innate immune memory, it would be conceivable to hypothesize that trained immunity response could be dysregulated by the dysbiosis in the elderly. Poor trained immunity response could render the elderly more susceptible to infections, while exuberant response might contribute to disease pathogenesis. However, more research is needed to understand how age-related changes in microbiota affect innate immune memory.

### Cross talk Between the Immune System and the Brain

Aging causes a great deal of deterioration in the central nervous system (CNS) through DNA damage, accumulation of waste products, oxidative stress, disturbed energy homeostasis, and impaired function [267]. The brain and the rest of the CNS are not immunologically isolated, as once thought: there is extensive cross talk between the immune system and the CNS. Brain homeostasis and regeneration depend on a robust immune system [268]. Therefore, deterioration of the immune system with old age contributes to and escalates brain aging and neurodegenerative diseases.

In the CNS parenchyma, the resident immune cell type is the microglia, which originates from primitive macrophage progenitors in the yolk sac early in development [269]. Microglia are extremely important for the maintenance of a healthy brain. They perform immunosurveillance, respond to infections, orchestrate the communication with the circulating immune system, regulate neurons, and other cell types in the brain, phagocytose cellular debris, misfolded proteins, toxic products, and even synapses [270]. Microglia are altered by aging and contribute to age-related neurodegenerative diseases [271]. Their phagocytic capacity is reduced with advancing age, and they contribute to a state of chronic low-grade inflammation. Due to this review’s focus on immune memory, we will not go into detail on microglia and instead focus on the role of adaptive immunity and trained immunity in the context of brain aging.

The blood–brain barrier (BBB) largely prevents the infiltration of immune cells into the brain. However, certain immune cell types are present in the cerebrospinal fluid (CSF) and the blood-CSF barrier at the choroid plexus (CP) [272]. CP, located in the brain’s ventricles, is a CSF-producing epithelial cell network with embedded capillaries. T cells are present in CP, and they regulate immune cell trafficking into CSF by IFNγ-dependent activation of CP epithelium [273].

Immune cells contribute to neuronal survival and neurogenesis during homeostasis, upon injury, or under neurodegenerative conditions [272]. Damage to the CNS induces a protective T cell response that prevents neuronal loss [274]. CD4^+^ lymphocytes play the most prominent role in this “neuroprotective immunity.”

#### Neuroprotective T Cell Immunity

CP harbors CD4^+^ T cells with an effector-memory phenotype that recognize CNS-specific self-antigens [275]. These cells can receive signals from circulation through the epithelium and the CNS through the CSF and orchestrate an integrated response to maintain brain homeostasis [276]. Astrocytes, a cell type that helps maintain synapses and the BBB, among various other functions, assume a neuroprotective phenotype and reduce neuronal apoptosis when co-cultured with T cells [277]. During spinal cord injury, CNS-specific autoreactive T cells migrate to the injury site, inhibit cyst formation, and contribute to the preservation of axons [278].

In T cell-deficient mice, the proliferation of progenitor cells is reduced, leading to lower numbers of new neurons, while neurogenesis is boosted in transgenic mice with excess CNS-specific autoreactive T cells [268]. Supplementation of the T-cell-derived cytokine IFNγ can enhance neurogenesis in old mice with Alzheimer’s disease [279]. CNS-specific T cells are also critical for spatial learning and memory. In immunodeficient mice, spatial memory is impaired but can be restored with reconstitution of immune cells even in aged mice [280]. In models of the motor neuron disease amyotrophic lateral sclerosis (ALS), T cell deficiency accelerates the disease, while reconstitution promotes neuroprotection and delays disease progression [281–283]. However, of note, T cells contribute to the death of dopaminergic neurons in mouse models of Parkinson’s disease [284].

One mechanism through which T cells improve brain maintenance is the regulation of brain-derived neurotrophic factor (BDNF). BDNF signaling via tropomyosin receptor kinase B (TrkB) plays wide-ranging roles, for example, in adult neurogenesis [285], memory formation and retrieval [286, 287], and is regulated by anti-depressant treatments [288]. BDNF levels are lower in T cell-deficient mice [268]. BDNF is associated with depressive behavior and immunization of mice with a myelin-derived peptide, generating CNS-specific immunity, restores BDNF levels, improves neurogenesis, and reduces depressive behavior [289]. Furthermore, healthy stress response in mice is associated with T cell trafficking in the brain and BDNF levels. Anxious behavior caused by stress is also reduced by immunization with a myelin-derived peptide [290]. Apart from neurons and microglia, T cells themselves are shown to secrete BDNF [291].

Tregs are also shown to be protective and delay disease progression in ALS by reducing microglial activation [292]. In models of Alzheimer’s disease, Treg transplantation enhances cognitive abilities and reduces amyloid plaques [293]. Moreover, a lower Treg/Th17 ratio is correlated with more severe disease in patients with multiple sclerosis, a debilitating autoimmune disease affecting neurons [294].

Although an over-exuberant immune response would impair brain function, a fine-tuned T cell immunity is clearly vital for healthy brain homeostasis and recovery from injury. Any intervention targeting this phenomenon must be carefully controlled to avoid inflammatory damage; however, the insights into adaptive immunity’s role in brain health open up new avenues to counter brain injury or age-related neurodegenerative diseases.

#### Trained Immunity in Microglia

Recent studies suggest that innate immune memory can be induced in microglial cells. One study found epigenetic reprogramming in microglia present for at least 6 months upon systemic LPS administration [295]. Interestingly, while a single LPS injection induced a trained phenotype in microglia, repeated LPS injection led to the induction of tolerance. Similarly, low-dose TNFα administration was also found to induce microglia training. In a mouse model of Alzheimer’s disease, trained immunity exacerbated the disease while tolerance alleviated it. A recent study confirmed the finding of LPS-induced training and demonstrated that systemic β-glucan administration could also induce trained immunity in microglia [296]. However, the trained phenotype of microglia was only observed two days after the priming and was no longer present at day 7, possibly indicating a lack of sustained epigenetic reprogramming. Therefore, it is worthwhile to investigate the strength and persistence of training with different doses and different injection regimens.

#### The Aging Brain

Many brain functions deteriorate with aging, with some even starting to decline after the third decade of life [297]. The impaired functions include processing speed, problem-solving, fluid reasoning, perceptual abilities, verbal fluency, and working memory. However, the impairments do not necessarily correlate with chronological age. It is rather an outcome of increased maintenance demand through the accumulation of damage and the inability of the immune system to monitor the brain to meet these demands. Of course, aging contributes to both the demand and the incapacity of the immune system through the mechanisms discussed earlier.

Aged microglia develop a pro-inflammatory phenotype [298]. Following a head injury or infection, they produce an excessive amount of pro-inflammatory cytokines for a longer time compared to a healthy young brain [299]. This inflammatory state leads to inhibited neurogenesis [300, 301]. A pro-inflammatory environment also inhibits modulators of long-term memory such as BDNF and activity-dependent cytoskeletal-associated protein and causes memory dysfunction [299]. Circulating BDNF levels decrease with age in humans, and brain levels are shown to decline in rodent models [302], which might reflect the age-associated drop in T cell numbers and function.

Aging is also associated with increased recruitment of effector memory CD8^+^ T cells to the CP and the meninges — the membranes covering the brain [303]. These cells were shown to impair microglial function during homeostasis but enhance pro-inflammatory cytokine production upon injury. Moreover, Treg numbers are elevated in elderly individuals; however, their migratory capacity and function are likely impaired since they are not able to control neurodegeneration. For instance, Tregs of multiple sclerosis patients have less immunosuppressive capacity and are unable to survive in sclerotic lesions in the brain [304].

In the case of chronic inflammation, while innate immune cells typically display tolerance leading to lower cytokine production, microglia acquire a primed to exhibit a more inflammatory phenotype, accelerating cognitive decline [305]. In addition, high levels of circulating TNFα observed in aged organisms might also cause damage by inducing trained immunity in microglia, as discussed above. Therefore, a well-balanced innate immunity is as essential for the healthy maintenance of the brain as adaptive immunity.

## Tackling Immune Aging From All Angles

**Fig. 2:**
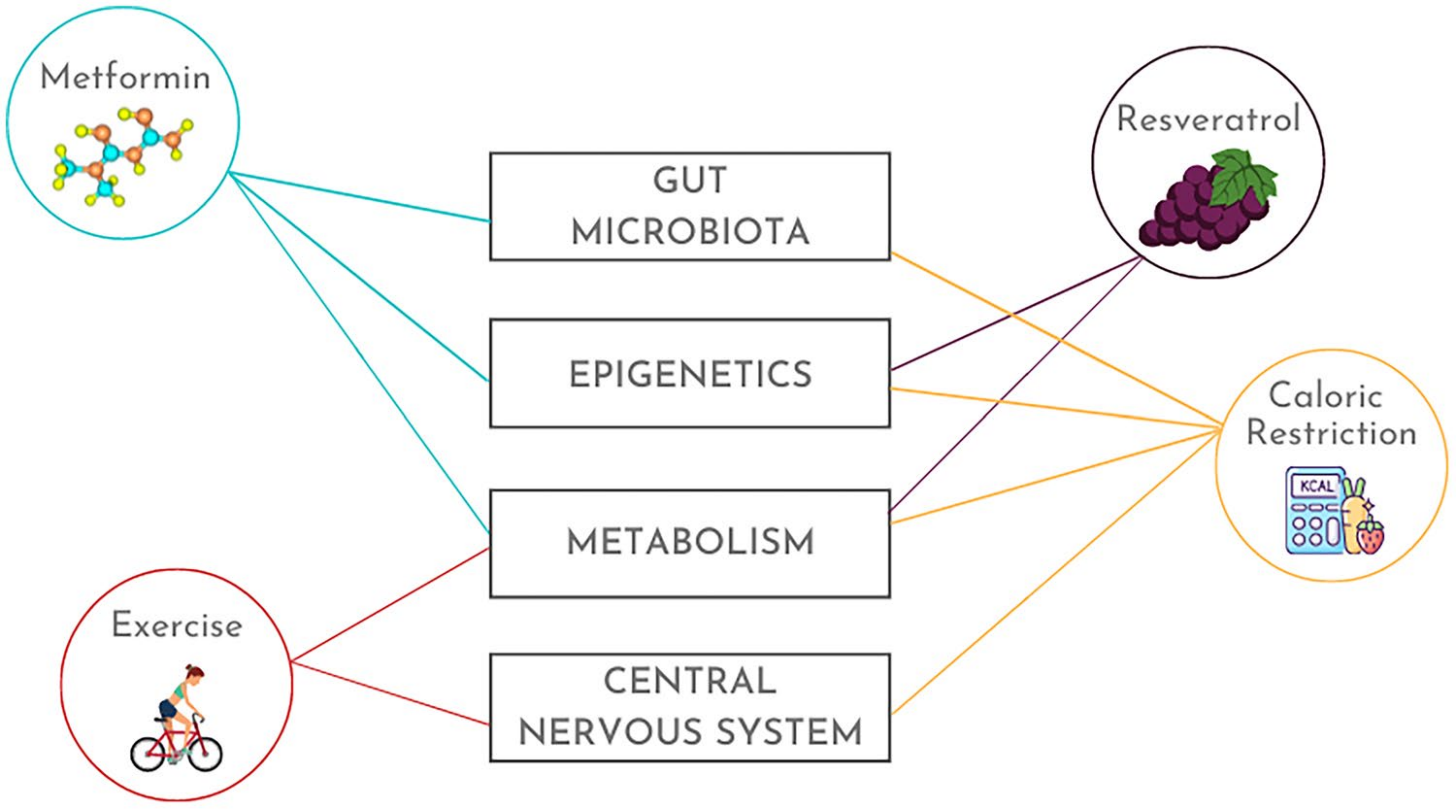
Promising anti-aging interventions that target multiple facets of the aging process. Metformin delays stem cell aging, improves mitochondrial function, prevents telomere shortening, reverses age-related epigenetic modifications, and reduces gut leakiness and dysbiosis. Physical exercise, even if initiated late in life, improves immune cell numbers and functions, restores mitochondrial metabolism, prevents cellular senescence, counteracts cognitive decline, and reduces risks for neurodegenerative diseases. Resveratrol, available in grapes and red wine, acts as an antioxidant, extends lifespan in various model organisms, attenuates systemic inflammation, and slows epigenetic aging. Caloric restriction by 20–40% enhances lifespan and reduces all-cause mortality in non-human primates, delays epigenetic aging, restores gut microbiota, and slows cognitive decline. Cellular mechanisms shared by these treatments include limitation of the mTOR/AKT axis and activation of AMPK and SIRT1

Efforts to slow or revert aging are far from scarce. However, the outcome measures assessed by most studies are restricted in the sense that they do not offer mechanistic insights or focus on specific processes. Yet, some exciting interventions, including caloric restriction, metformin, and physical exercise, interfere with aging on multiple levels encompassing immunity, metabolism, epigenetics, microbiota, and the nervous system (Fig. 2). The following chapters discuss different ways to tackle the aging problem and detail the mechanisms of the most promising anti-aging treatments.

### Metabolic Interventions

For most of the human evolution, nutrients were scarce, and a great deal of physical activity was required to obtain them. Thus, humans evolved to adapt to those conditions. Our current sedentary lifestyle with an overabundance of nutrients is proposed to cause the high prevalence of metabolic diseases, such as obesity, diabetes, and cardiovascular disease [306]. Furthermore, age is a risk factor for these conditions, as mentioned before, and immunosenescence has a lot in common with metabolic disease profiles. Therefore, focusing on metabolic interventions is a sensible approach to tackle aging and metabolic disorders simultaneously. Caloric restriction (CR) and exercise, bringing us closer to the ancestral conditions, take the lead in this line of research.

CR refers to a reduction of total calory intake by 20–40%. From yeasts to non-primates, CR has repeatedly been shown to enhance lifespan [307]. In rhesus monkeys, CR starting from young adulthood reduced the risk of mortality related to age-related causes by threefold and all-cause mortality by 1.8-fold [308]. In another study, CR decreased the incidence of diabetes, cancer, and cardiovascular disease while also delaying disease onset [309]. A contrasting study reported no improvement in survival, although the incidence of cancer and diabetes was reduced [310].

In a randomized controlled trial of 218 non-obese people, a 2-year CR diet reduced circulating TNFα levels and strikingly decreased cardiometabolic risk markers, such as cholesterol and triglycerides, without any intervention-related adverse effects [311]. So far, there is no human study reporting a significant effect of CR on longevity. Large and extensive studies with genetically diverse populations are needed to solidify the promise of CR in humans.

Various metabolic impacts of CR include downregulation of mTOR and insulin signaling and activation of SIRT1, which all have broad implications on immune cell function [312]. CR is shown to delay T cell senescence in rhesus monkeys [313]. Furthermore, CD4^+^ and CD8^+^ naïve T cell pools were expanded, and thymic output and T cell proliferation were increased, but IFNγ production by CD8 + cells was reduced after CR. Although reducing the amount of calories taken seems to reverse age-induced metabolic changes and improve health and longevity, it is important to note that a few studies in rodents reported an impaired adaptive response and increased mortality against influenza A and West Nile viruses in elderly animals after CR [314, 315]. However, a recent mouse study revealed protective effects of CR against *M. tuberculosis* infection. This effect was related to metabolic shift characterized by mTOR inhibition but enhanced glycolysis and reduced FAO, along with increased autophagy [316]. mTOR inhibitor rapamycin acted synergistically with CR and further enhanced autophagy, leading to more efficient inhibition of *M. tuberculosis*.

Similar to CR, exercise is promising to interfere with immunosenescence. Regularly exercising older women had better NK and T cell functions compared to age-matched sedentary women [317]. Naïve T cell numbers and thymic output were higher in physically active elderly, similar to young adults, compared to sedentary ones [318]. They also had lower circulating IL-6 and higher IL-7, which is essential for T cell development. However, senescent CD8^+^ T cell numbers did not differ between groups. After an 8-week training program, immune cells of elderly adults displayed enhanced autophagy and downregulated NLRP3 inflammasome [319]. Exercise also improved mitophagy and mitochondrial biogenesis in skeletal muscle cells and immune cells alike, restoring the cellular metabolic status impaired by aging [320].

Apart from lifestyle interventions, chemical metabolic regulators are also investigated for their anti-aging potential. Metformin, safely used in humans for more than 60 years for its glucose-lowering effect, attenuates age-associated hallmarks through a plethora of mechanisms. These include activation of AMPK, inhibition of mTORC1, improved mitochondrial biogenesis, downregulation of insulin/IGF1 signaling, and activation of SIRT1 [321]. Furthermore, metformin delays stem cell aging and reduces telomere shortening. Overall, it seems to act on all hallmarks of aging. A large clinical trial of more than 3000 individuals aged 65–79 is currently being planned to assess the anti-aging potential of metformin (https://www.afar.org/tame-trial).

Everolimus, another mTOR inhibitor, attenuated immunosenescence and improved antibody responses to influenza vaccination in the elderly [322]. Even though most immune cell subsets were not altered in this study, T cells positive for programmed cell death protein 1 (PD-1), a marker of exhaustion, were markedly reduced. A follow-up study with 264 elderly subjects reported upregulated antiviral expression, improved response to influenza vaccination, and overall fewer infections [323].

SIRT1 activation is another approach to tackle immunosenescence. It is known to improve B cell proliferation and function, and therefore could help improve antibody responses declining with age [324]. SIRT1 can modulate metabolic pathways through protein and histone deacetylation [325]. Targets of SIRT1 include NF-κB, hypoxia-inducible factor 1-alpha (HIF1α), and FOXO transcription factors. Moreover, SIRT1 activation potentiates BCG-induced trained immunity response [326]. Despite mouse studies with SIRT1-activators showing delayed age-related phenotypes and increased lifespan [327, 328], there is no evidence suggesting that SIRT1 is associated with longevity in humans [329].

Resveratrol, a polyphenol compound found in red wine, is a potent activator of SIRT1 [330]. It is also shown to activate AMPK, therefore repressing mTOR signaling [331]. Apart from in vitro studies and inflammatory disease models displaying resveratrol’s antioxidant and anti-inflammatory activity [332], several mice studies reveal its antiviral capacity [333, 334]. In terms of longevity, studies failed to report a significant lifespan extension by resveratrol in healthy mice [327, 335]. However, in mice fed with a high-calorie diet, resveratrol shifted the transcriptional profile towards that of standard-fed mice [336]. It also improved insulin sensitivity and increased survival. Similar results were observed in rhesus monkeys on a high-fat, high-sugar diet [337]. Thirty-day supplementation of obese men with resveratrol induced metabolic changes through the AMPK-SIRT1 axis and reduced systemic inflammation, glucose, and triglyceride levels [338]. However, a similar study did not report any beneficial effects of resveratrol [339].

Overall, there are highly promising therapeutic approaches targeting metabolic pathways underlying immunosenescence and age-associated metabolic diseases. However, large-scale randomized control trials in humans are needed to see whether these exciting observations in non-human primates and smaller model organisms are translatable for human use.

### Strategies Modulating Epigenetics

Epigenetic interventions have been employed for several age-related diseases, e.g., cancer, diabetes, and Alzheimer’s disease; however, only a few studies specifically target age-dependent changes in the epigenetic structure [340]. Instead, metabolic interventions employed to halt immunoaging also work by altering the age-associated epigenetic landscape. Resveratrol, CR, and metformin are three promising therapy options reconfiguring age-related DNA methylation and histone modifications in the elderly.

An intriguing study revealed that regenerating the thymus resulted in a 2.5-year younger epigenetic age [341]. Participants between 51 and 65 years of age received a 1-year treatment with recombinant human growth hormone, dehydroepiandrosterone (DHEA), which is a steroid hormone precursor, and metformin. The treatment led to restored functional thymic mass, changes in the immune cell subsets, and cytokine production, as well as altered epigenetic profile, which was associated with younger age.

Rhesus monkeys, who were exposed to 40% caloric restriction, were late to display the methylation changes found in the older monkeys [342]. Although this study does not provide direct evidence of a longer lifespan associated with delayed methylation drift, it suggests that CR could be used to slow down the aging process. In line with this, improving the lifespan of mice with resveratrol or CR resulted in slower epigenetic aging [343]. Life-long CR has also shown to prevent age-related DNA methylation changes in the brain, providing neuroprotection [344].

A few studies explain how CR could affect epigenetics. These mechanisms include decreased histone acetylation mediated by increased *SIRT1* expression, higher DNA methyltransferase (DNMT) activity, and hypermethylation of specific regulatory genes, such as Ras [340]. Similarly, metformin acts on epigenetic marks via activating SIRT1 and inhibiting HDACs [345]. To our knowledge, there is no research investigating the effects of CR on aging-related epigenetic alterations, possibly due to the limitations of implementing such long-term interventions on humans.

### Potential Treatments Targeting Microbiota

Since gut microbiota regulates host metabolism, anti-aging interventions targeting metabolism inevitably affect the gut microbiota. As an example, besides acting on metabolic pathways, metformin modulates the gut microbiota. A study investigating the effects of metformin in obese and aged mice found a decrease in IL-1β and IL-6 in the epididymal fat, which was associated with changes in the gut microbes [346]. Furthermore, type 2 diabetes patients who take metformin had a higher abundance of *Akkermansia* in their guts [347], which was correlated with lower bacterial translocation and risk of dysbiosis [348]. In line with these, metformin reduced age-related leaky gut and inflammation in mice [349].

Another treatment strategy to halt immunoaging by targeting the microbiota is the use of pro- and prebiotics. Probiotics are supplements containing live microorganisms, while prebiotics is substrates that microorganisms can utilize for a living [350]. Although there is conflicting evidence, studies suggest that regular probiotics use can modulate the diversity and abundance of the gut microbes, decreasing the incidence of dysbiosis [351, 352]. Probiotics are associated with improved immune responses evident from increased B and T cell counts, enhanced NK cell activity [353] and higher IgA production against influenza virus in older individuals [354]. Furthermore, supplementation with probiotics helped reduce the growth of opportunistic bacteria *Clostridium difficile* among the elderly [355]. Contrary to these findings, a meta-analysis of 10 randomized controlled studies showed no beneficial effect of probiotics on decreasing inflammatory cytokine production [356].

The combination of probiotics with prebiotics, i.e., synbiotics, also has beneficial effects, like probiotics supplementation. Two months of treatment in elderly individuals with a synbiotic formula significantly improved the metabolic syndrome parameters in circulation and decreased inflammatory proteins, such as TNFα and C-reactive protein [357]. A double-blind 4-week symbiotic treatment study reported an increase in *Bifidobacteria*, *Actinobacteria*, *Firmicutes*, and the metabolite butyrate in the treatment group compared to placebo, while *Proteobacteria* and pro-inflammatory cytokines were lower [358].

Caloric restriction could be another treatment strategy to improve cognitive functions, metabolic parameters, and gut microbiota in the elderly. CR slowed the cognitive decline in a mouse model of Alzheimer’s disease, associated with increased *Bacteroides* in the guts. Aged mice receiving 30% fewer calories for 2 months displayed significant shifts in their microbiota towards a more balanced composition similar to that of young mice [359]. Lifelong CR induced more extensive changes in the microbiota, reduced the concentration of inflammatory peptides, and increased the lifespan of mice [360]. However, a recent study revealed that severe CR, more than 50%, disrupts the diversity of microbiota and leads to the growth of pathogenic bacteria *C. difficile* [361]. Thus, it is critical to carefully determine the extent and duration of CR.

### Interventions for Brain Aging

Physical exercise is an excellent way of promoting brain health. Exercise counteracts cognitive impairment, reduces dementia risk, improves spatial memory, and enhances neuroplasticity [362]. Physical activity can attenuate the effects of risk alleles for memory impairment [363] and protect against the development of Alzheimer’s disease [364, 365]. A systematic review of 16 studies with a total of 163,797 participants reported that regular exercise led to 28% and 45% risk reduction in dementia and Alzheimer’s, respectively [366]. Of note, exercise-associated risk reduction was observed in most of the individual studies irrespective of the frequency and intensity of the exercise.

Studies suggest antioxidant and anti-inflammatory effects of exercise as potential mechanisms behind neuroprotection [367, 368]. Anti-inflammatory consequences of exercise include reduced circulating IL-6 but increased IL-10 and IL-1RA, lower numbers of Treg, and higher numbers of inflammatory monocytes in circulation, and inhibited monocyte function [369]. Besides these, physical exercise is associated with reduced senescent T cells, increased NK cell cytotoxicity and neutrophil phagocytosis, and longer telomeres in leukocytes [370]. Additionally, moderate cardiovascular exercise improved seroprotection after influenza vaccination in the elderly [371]. Slowing down immunosenescence would limit brain aging and cognitive decline through improved immunosurveillance and repair of the CNS.

Moreover, even a single exercise session increases BDNF levels which is further enhanced with regular exercise [372]. Interestingly, the exercise-related increase in BDNF is more pronounced in males compared to females. Ketone bodies are also shown to induce *BDNF* expression [373, 374], possibly contributing to the neuroprotective effect of ketogenic diets in neurological diseases [375].

CR is another intervention shown to prevent neuronal damage. It leads to increased BDNF expression and enhanced neurogenesis [376], causes an energetic shift from glycolysis to the use of ketone bodies, protects white matter integrity, and improves long-term memory in mice [377]. In rats, an alternate-day CR regimen promotes neuronal resistance to chemically induced damage [378]. One mechanism of CR-induced neuroprotection is likely due to the suppression of oxidative stress in the brain [379, 380]. However, severe CR with 50% reduction of calorie intake was reported to cause depressive behavior in rats [381]. In mouse models of Alzheimer’s disease, CR is able to limit amyloid plaque deposition [382, 383], possibly through a mechanism involving SIRT1 activation [384].

Despite all the positive results in rodents, neuroprotective effects of CR are not very clear in non-human primates, while large human studies are lacking [385]. Nevertheless, a small randomized controlled trial with humans resulted in no significant improvement in cognitive function [386]. Another clinical study on older adults showed improved memory scores upon 3 months of CR [387]. Improved memory, along with higher functional connectivity in the hippocampus, was reported in obese women that underwent a 3-month CR diet [388]. More extensive human studies with CR are necessary to understand the extent of the neuroprotective effects.

Interestingly, BCG vaccination was recently shown to reduce the risk of Alzheimer’s and Parkinson’s diseases in bladder cancer patients treated with BCG immunotherapy, compared to non-treated patients [389, 390]. In bladder cancer treatment, BCG is applied directly into the bladder, rather than the usual intradermal route of administration. Exciting future research projects would be assessing the effects of intradermal BCG on neurodegenerative diseases and investigating the underlying mechanisms to find out if trained immunity plays a role in the neuroprotective effects. Currently, a clinical trial is underway using intradermal BCG injections in late-onset Alzheimer’s patients (NCT04449926).

## Concluding Remarks

Biological aging is a complex process involving all systems of the organism. The immune system is at the very center of it, interacting with all the others. The aging immune system is a culprit for the high susceptibility of the elderly to infections and age-related metabolic and neurodegenerative diseases, among others. Therefore, improving innate and adaptive immunological responses is immensely important to reduce infection-related morbidity and mortality and enhance vaccine responsiveness in older individuals. Here, we also presented a large body of research hinting towards new roles of immune memory in metabolic regulation and maintaining a healthy central nervous system. Approaching aging from all angles, with immunity as a central node, and designing anti-aging interventions targeting the common mechanisms ubiquitously affected by aging is a sensible way to further research. Behavioral interventions such as caloric restriction and physical exercise as well as pharmacological agents such as metformin and resveratrol are able to regulate many facets of aging and have yielded promising results in animal models and humans. A comprehensive strategy is essential for human beings striving to lead long lives with healthy guts, functional brains, and free of severe infections.
